# Treatment patterns and economic burden of sickle-cell disease patients prescribed hydroxyurea: a retrospective claims-based study

**DOI:** 10.1186/s12955-019-1225-7

**Published:** 2019-10-16

**Authors:** Nirmish Shah, Menaka Bhor, Lin Xie, Rashid Halloway, Steve Arcona, Jincy Paulose, Huseyin Yuce

**Affiliations:** 1 Duke University, Durham, NC USA; 2 Novartis Pharmaceuticals Corporation, East Hanover, NJ USA; 3 STAinMED Research, Ann Arbor, MI USA; 4 New York City College of Technology (CUNY), New York, NY USA

**Keywords:** Hydroxyurea, Adherence, Discontinuation, Health care, Cost, Utilization

## Abstract

**Background:**

This study aimed to evaluate sickle-cell disease (SCD) treatment patterns and economic burden among patients prescribed hydroxyurea (HU) in the US, through claims data.

**Methods:**

SCD patients with pharmacy claims for HU were selected from the Medicaid Analytic Extracts (MAX) from January 1, 2009 - December 31, 2013. The first HU prescription during the identification period was defined as the index date and patients were required to have had continuous medical and pharmacy benefits for ≥6 months baseline and 12 months follow-up periods. Patient demographics, clinical characteristics, treatment patterns, health care utilization, and costs were examined, and variables were analyzed descriptively.

**Results:**

A total of 3999 SCD patients prescribed HU were included; the mean age was 19.24 years, most patients were African American (73.3%), and the mean Charlson comorbidity index (CCI) score was 0.6. Asthma (20.3%), acute chest syndrome (15.6%), and infectious and parasitic diseases (20%) were the most prevalent comorbidities. During the 12-month follow-up period, 58.9% (*N* = 2357) of patients discontinued HU medication. The mean medication possession ratio (MPR) was 0.52, and 22.3% of patients had MPR ≥80%. The average length of stay (LOS) for SCD-related hospitalization was 13.35 days; 64% of patients had ≥1 SCD-related hospitalization. The mean annual total SCD-related costs per patient were $27,779, mostly inpatient costs ($20,128).

**Conclusions:**

Overall, the study showed the patients had significant unmet needs manifest as poor medication adherence, high treatment discontinuation rates, and high economic burden.

## Background

Sickle-cell disease (SCD) consists of a group of rare genetic blood disorders characterized by a single missense mutation (Glu6Val) in the β-globin gene. The mutated hemoglobin in SCD, known as sickle hemoglobin (HbS), is less soluble and prone to polymerization upon deoxygenation, causing red blood cell sickling, which contributes to numerous other complications [1–3], as SCD progresses early on into a systemic disease. Vaso-occlusion is the primary indicator of SCD and can lead to serious acute and chronic complications; vascular dysfunction, inflammation, and P-selectin mediated cell-to-cell and cell-to-endothelium adhesion play an important role in the pathophysiology of SCD vaso-occlusive crisis (VOC), which is the most common clinical manifestation of SCD. Every VOC increases morbidity, and can result in organ damage or failure, acute chest syndrome (ACS), stroke, end-organ damage, or death [4–12]. The Centers for Disease Control and Prevention has estimated that 100,000 Americans are burdened with SCD, and in newborn infants it occurs most frequently among African-Americans [13]. Most notably, SCD has been associated with high physical and economic burden [14].

Among SCD-related complications, VOC events have long been identified as a higher risk factor for death and the most common cause of hospital admission among SCD patients [15]. A 5-year study carried out using Florida Medicaid program data estimated that SCD patients had an average of 3.7 inpatient hospitalizations and 24.1 hospital days during the study period, with roughly 84% attributable to SCD-related diagnoses [16].

Currently, treatment and prevention options for VOCs are limited. It was not until the 1990s that the efficacy of hydroxyurea (HU) was first demonstrated in the prevention of VOCs [17]. In the US, HU was first approved by the FDA for use in the adult SCD population in 1998, and was recently approved for use in the pediatric SCD population in 2017 [18]. HU affects certain cells in the body, such as cancer cells and sickled red blood cells. It is used to treat cancers including chronic myeloid leukemia, ovarian cancer, and certain types of skin cancer such as squamous cell cancer of the head and neck. HU is also used palliatively to reduce pain episodes and the need for blood transfusions in people with sickle cell anemia, although it will not cure sickle cell anemia [19]. HU’s mechanism of action (MOA) includes increasing the concentration of fetal hemoglobin, lowering the number of circulating leukocytes and reticulocytes, and decreasing their expression of adhesion molecules, thereby decreasing vascular occlusion. Other MOAs include increasing the size of the reticulocytes and improving cellular deformability, which increases blood flow and reduces vaso-occlusion and its associated complications. Furthermore, metabolism of HU releases nitric oxide, which can cause local vasodilation [20, 21]. HU has been proven to effectively decrease the frequency of pain episodes and other acute complications in multiple randomized controlled trials [22]. Studies have shown that HU can be used as a substitute for chronic transfusions for the prevention of primary stroke among high-risk SCD pediatric patients with abnormal transcranial doppler (TCD) flow velocity [23, 24]. Also, observational studies have shown a relationship between HU use and decreased rates of hospitalization and blood transfusions [25, 26]. More recently, the FDA approved oral L-glutamine therapy for SCD patients aged 5 years and older to reduce the number of acute complications associated with the blood disorder [27]; while this medication’s MOA is not well understood, it is believed to result from a reduction of intracellular oxidation damage [28]. Adverse events associated with HU include GI symptoms such as loss of appetite, nausea, constipation and diarrhea; other symptoms include infection and bleeding [19].

VOCs lead to significant health care utilization and are the most common cause of emergency room (ER) visits and hospital admissions among SCD patients, with total medical costs exceeding $1.1 billion in the US annually [16]. A US study using the Healthcare Cost and Utilization Project (HCUP) database was conducted to assess nationwide hospitalizations for SCD patients from 1994—2004, and estimated the annual direct hospitalization-related costs for SCD at $488 million [29]. Another study in 2015 estimated the average cost per patient-month at $1389, with a lifetime cost of care of approximately $460,000 per patient with SCD [16]. The Bou-Maroun et al. analysis in 2018 determined that annual health care expenditures for SCD hospitalization resulted in costs over $900 million, with a median hospitalization cost of $14,337 per stay per patient [30]. Blinder et al. examined age-related treatment patterns among SCD patients and the associated complications and health care costs; their results showed that quarterly total health care costs per patient ranged from $11,913–$11,957 among patients who received ≥10 blood transfusions [31].

Several studies that have examined the cost-effectiveness of HU support the advantages of HU use for clinical and economic benefits [26]. One study observed that HU use among pediatric patients resulted in reduced hospitalization and decreased health care costs [32]. Notably, studies have also shown that there is underutilization of HU among SCD patients, considering the significant efficacy of the drug [33]. However, few analyses have examined HU treatment patterns among SCD patients, and none have examined HU treatment patterns in a real-world setting. To demonstrate real-world treatment patterns of SCD patients prescribed HU, this study descriptively evaluated treatment patterns and the economic burden of SCD patients who were prescribed HU in the US Medicaid database population.

## Methods

### Data source

This was a retrospective, descriptive study of the characteristics and treatment patterns of SCD patients prescribed HU during the period of 01JAN2009 through 31DEC2013, using the US Medicaid population database.

The MAX data system contains extensive individual-level information on the characteristics of Medicaid enrollees in all 50 states and the District of Columbia, as well as the services used during a calendar year. Specifically, MAX consists of 1 personal summary file and 4 claims files that provide fee-for-service (FFS) claims, managed care encounter data, and premium payments. The study included FFS patients from all available states and managed care enrollees who resided in 14 states with the most relatively complete data available: Arizona, California, Indiana, Kansas, Kentucky, Minnesota, Nebraska, New Jersey, New Mexico, New York, Oregon, Tennessee, Texas, and Virginia. Service use among Managed Care enrollees is captured in encounter data. Patients who had dual eligibility with Medicare were not included in this study due to incomplete information in the MAX database.

### Patient selection

Patients were included if they had ≥1 pharmacy claim for HU during the identification period (01JUL2009 -01DEC2012); the first observed HU claim date was designated as the index date.

Patients were also required to have continuous health plan enrollment with medical and pharmacy benefits during the 6 months before the index date (baseline period) and 12 months after the index date (follow-up period). In addition, they were required to have ≥1 diagnosis claim with SCD (International Classification of Diseases, Ninth Revision, Clinical Modification [ICD-9-CM] codes: 282.41–282.42, 282.60–282.69) before the index date. Patients were excluded from the study if they were enrolled in a clinical trial during the study period (identified using ICD-9-CM: V70.7).

### Baseline measures

Socio-demographics and clinical characteristics were evaluated for the baseline period including age, sex, race/ethnicity, geographical region, and Charlson Comorbidity Index (CCI) score. Baseline individual comorbid conditions were flagged including VOCs, pulmonary conditions (eg, ACS), cerebrovascular conditions (eg, stroke), hepatic and biliary conditions (eg, gallstones), splenic conditions (eg, splenic sequestration), and other conditions that commonly occur among SCD patients. Baseline all-cause health care resource utilization and costs were also identified by inpatient, outpatient (ER, office, other), and pharmacy visits. Health care costs were calculated only for patients enrolled in an FFS Medicaid plan.

### Outcome measures

HU treatment patterns during the 12-month follow-up period were examined. HU discontinuation was defined as an observed refill gap of ≥90 days between two subsequent prescriptions. The period from the index date to the discontinuation date was also examined. Sensitivity analysis using 30- and 60-day refill gaps were also conducted to measure discontinuation. Medication possession ratio (MPR) was calculated as the ratio of the total number of days of supply of HU to the total number of days in the follow-up period. The average daily dose was examined; dosing modification between the average HU daily dose during the follow-up period and on the index date was calculated. Treatments prescribed during the first 12 months of the follow-up period were identified. Monitoring patterns such as laboratory and radiology tests—in addition to all-cause and SCD-related health care resource utilizations and costs during the 1-year post-index date—were examined by facility type. The rate of complicated and uncomplicated VOCs was also examined, and health care costs were calculated only for patients enrolled in an FFS Medicaid plan.

### Statistical methods

All variables were analyzed descriptively. Percentages and numbers were provided for dichotomous and polychotomous variables. Means and standard deviations were examined for continuous variables.

## Results

### Baseline characteristics for the SCD patients prescribed HU

**Fig. 1:**
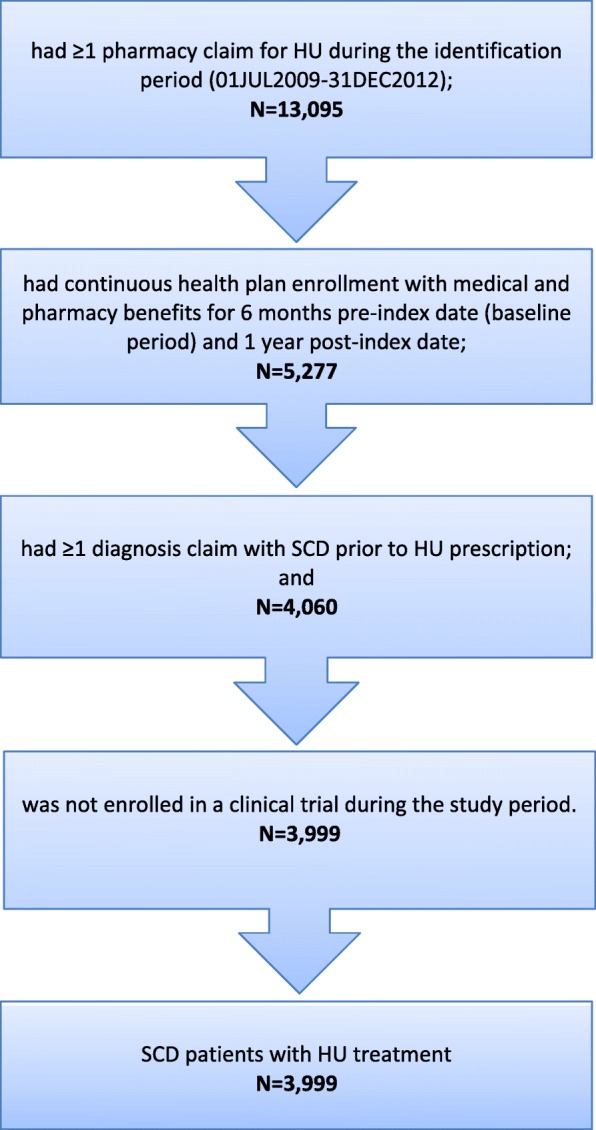
Flow chart for patient selection criteria. HU: hydroxyurea; SCD: sickle cell disease

A total of 3999 eligible patients met the study selection criteria and were included for analysis (Fig. 1). The mean age was 19.24 years (standard deviation [SD] = 11.85). Approximately half the study population (51.8%) were aged under 18 years, and the majority (73.3%) of SCD patients were African-American. The remaining 26.7% were comprised of whites, Hispanics, and patients of other or unspecified ethnicity. In addition, the mean CCI score was 0.60 (Table 1).

**Table 1:** Baseline demographic and clinical characteristics of SCD patients prescribed HU

Patient characteristics	Sickle cell patients prescribed HU	
	(*N* = 3999)	
	N/Mean	%/SD
Age (years)	19.24	11.85
Age Group		
< 2	26	0.7%
2–5	327	8.2%
6–11	786	19.7%
12–17	933	23.3%
18–30	1272	31.8%
31–45	501	12.5%
≥ 46	154	3.9%
Sex		
Male	2033	50.8%
Female	1966	49.2%
Race/Ethnicity		
White	111	2.8%
Black	2933	73.3%
Hispanic	296	7.4%
Other	33	0.8%
Unknown	626	15.7%
Geographic Region		
Northeast	1335	33.4%
North Central	590	14.8%
South	1501	37.5%
West	573	14.3%
Charlson Comorbidity Index Score	0.60	0.99
0	2447	61.2%
1	1035	25.9%
2–3	440	11.0%
4+	77	1.9%
Individual Comorbid Conditions (≥5%)		
Fever	1255	31.4%
Asthma	811	20.3%
Infectious and parasitic diseases	798	20.0%
Acute chest syndrome	623	15.6%
Constipation	485	12.1%
Upper respiratory tract infections	462	11.6%
Aseptic (avascular) bone necrosis	307	7.7%
Iron overload	298	7.5%
Gallstones	214	5.4%
Chronic pain	205	5.1%
Neoplasms benign and malignant	198	5.0%
Sepsis	198	5.0%

*HU* hydroxyurea, *SCD* sickle cell disease, *SD* standard deviation

More than half of the study population (53.6%) had a VOC during the baseline period. Pulmonary conditions such as asthma (20.3%), ACS (15.6%), and upper respiratory tract infections (11.6%) were the most prevalent comorbid conditions. Other frequent conditions observed among the study population included fever (31.4%), infectious and parasitic diseases (20.0%), and constipation (12.1%, Table 1).

### Baseline health care utilization

**Fig. 2:**
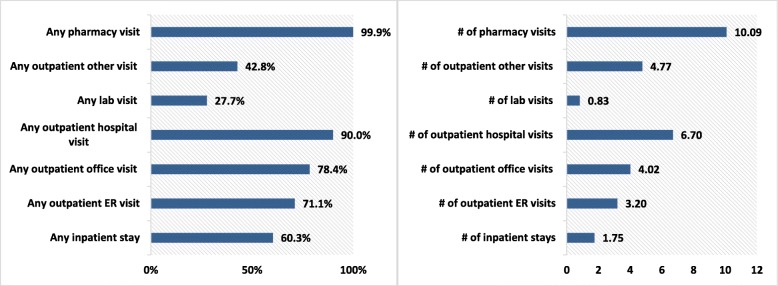
Baseline all-cause health care resource utilization. ER: emergency room

During the 6-month baseline period, all-cause health care utilization results showed that 90.0% had ≥1 outpatient hospital visit, 78.4% had ≥1 outpatient office visit, 71.1% had ≥1 outpatient ER visit, and 60.3% had ≥1 inpatient visit. The average length of stay (LOS) was 9 days, with a mean number of inpatient stays of 1.75 and 3.20 for outpatient ER visits (Fig. 2).

### Baseline health care costs

Mean total costs for all-cause health care during the 6-month baseline period were $19,194; the primary cost drivers were inpatient ($12,806); pharmacy ($2371); outpatient ($3563); and ER costs ($406); other costs including laboratory visits and ambulatory costs amounted to $48.

### Follow-up results

#### Treatment patterns for SCD patients prescribed HU

**Fig. 3:**
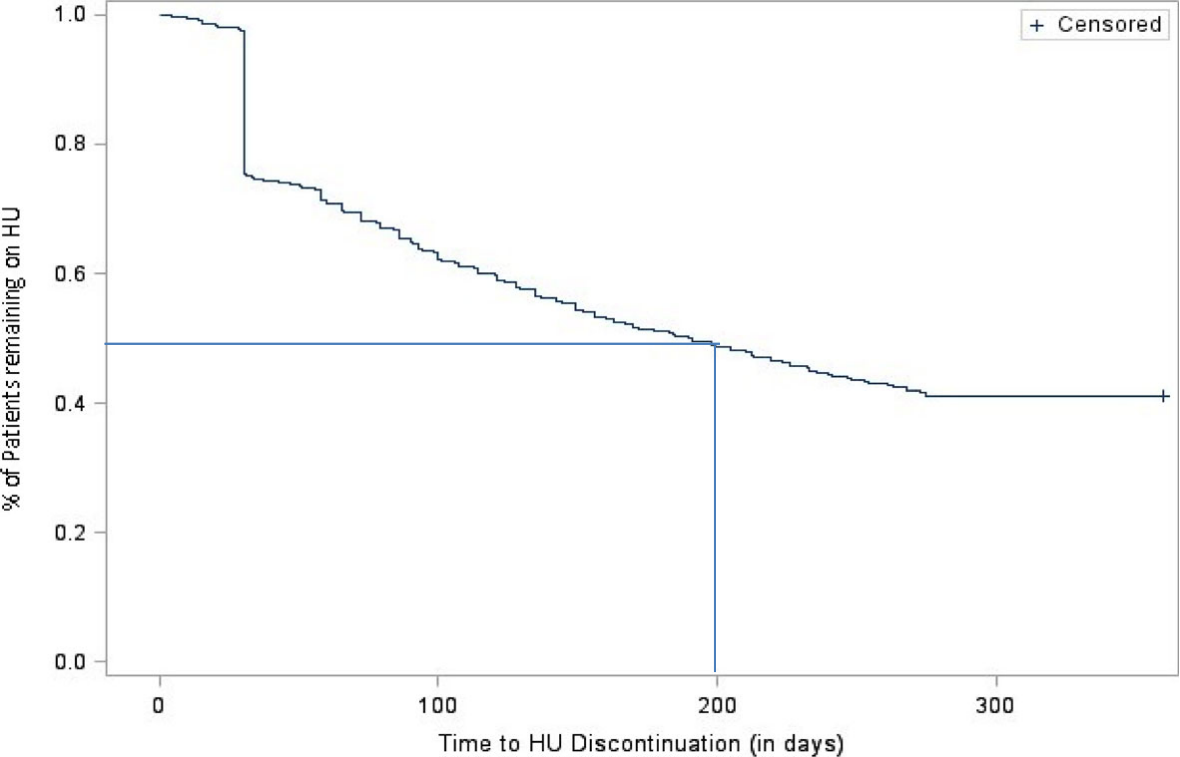
Kaplan Meier curve for time to HU discontinuation. HU: hydroxyurea

Treatment patterns during the 12-month follow-up period were measured. Using a 90-day treatment gap to define discontinuation, the rate of HU discontinuation was 58.9% (*N* = 2357). The average time-to-discontinuation was 202 days, with and < 50% of the SCD patients remaining on HU after 200 days (Fig. 3). During the 1-year follow-up period, 52.5% of the 2357 patients reinitiated HU. Using a 30-day gap to define discontinuation, the rate of HU discontinuation was 87.8% (*N* = 3512), and 76.7% (*N* = 2692) reinitiated HU. With a 60-day gap, 72% (*N* = 2878) of patients discontinued HU, and 65% (*N* = 1870) reinitiated HU (Additional file 1: Table S1).

**Fig. 4:**
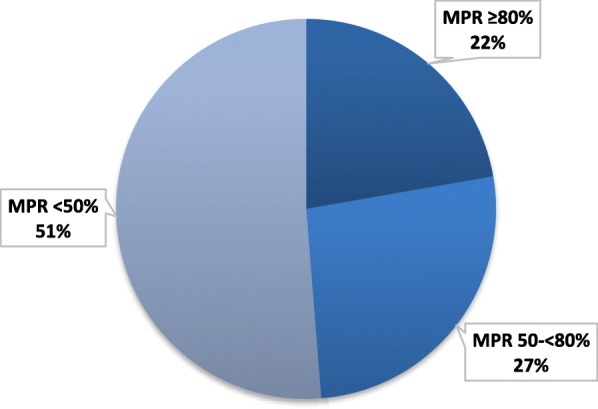
MPR for SCD patients during the 12-month follow-up period. MPR: mean possession ratio

The mean MPR was 0.52 (SD = 0.36) among the overall HU patients; less than half (48.7%) of the study population had MPR ≥50%, and less than one-quarter (22.3%) of patients had MPR ≥80% (Fig. 4).

**Fig. 5:**
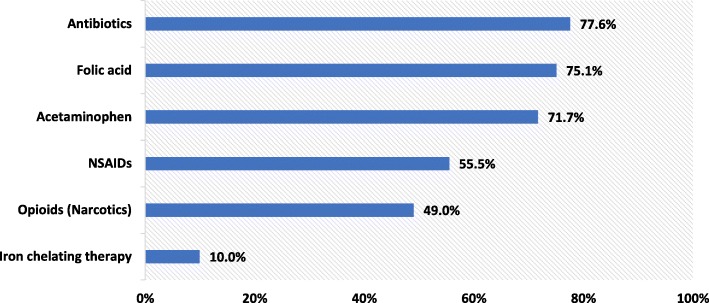
Concomitant SCD treatments during the follow-up period. SCD: sickle cell disease; NSAIDs: non-steroidal anti-inflammatory drugs

The average index dose of HU was 980.6 mg with an average decrease of 3.9 mg during the follow-up period. Other concomitant medications used after the index date included antibiotics (77.6%), folic acid (75.1%), and opioids (49%; Fig. 5). Nearly 38.1% of the population had ≥1 episode of blood transfusion with an average of 1.11 blood transfusions during the follow-up period; 25.0% were administered transcranial doppler ultrasonography. Only 4.4% of the population had pneumococcal vaccine, 2.9% had meningococcal vaccine, and 0.3% of the patients received a bone marrow transplant during the 12-month follow-up period (Table 2).

**Table 2:** Treatment patterns of SCD patients prescribed HU

Outcomes	SCD patients prescribed HU therapy	
	(*N* = 3999)	
	N/Mean	%/SD
HU Dose		
HU index dose (mg)	980.63	656.62
Dosing modification	−3.85	468.16
SCD Management (during the first 12 months)		
Blood transfusions		
# of Patients	1523	38.1%
Number of blood transfusions	1.11	2.28
Transcranial Doppler ultrasonography	1001	25.0%
Pneumococcal vaccine	175	4.4%
Meningococcal vaccine	115	2.9%
Bone marrow transplant	11	0.3%
Monitoring Patterns (during a 12-month period)		
Chest X-ray	2784	69.6%
X-ray of extremity	839	21.0%
Abdominal plain film	586	14.7%
Computerized tomography	341	8.5%
Nuclear medicine studies	158	4.0%
Echocardiography	1239	31.0%
Electrocardiogram	1444	36.1%
Eye exams	909	22.7%
Complete blood count	2945	73.6%
Iron tests	1167	29.2%

*SCD* sickle cell disease, *HU* hydroxyurea, *SD* standard deviation

#### Monitoring patterns

A majority (73.6%) of the patients had a complete blood count performed, 69.6% had a chest x-ray, 36.1% had an electrocardiogram, 31% had echocardiography, 29.2% had serum iron studies done, and 21% had an x-ray of an extremity carried out during the follow-up period. Other tests carried out included eye examination (22.7%), plain abdominal x-ray (14.7%), and computerized tomography (CT) scan (8.5%) during the follow-up period (Table 2). Approximately 99.1% of the patients who had a VOC presented with a form of complication, and 10.13% of the patients had ACS during the follow-up period.

#### Health care resource utilization

**Fig. 6:**
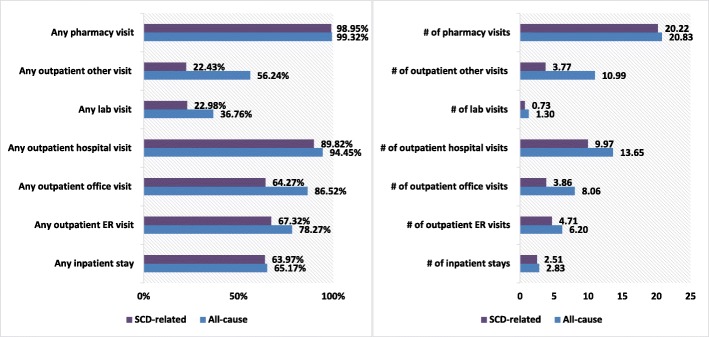
Proportion of patients with numbers of visits for all-cause and SCD-related health care utilization. SCD: sickle cell disease; ER: emergency room

All-cause health care resource utilization results showed 65.2% of patients had ≥1 inpatient visit during the follow-up period. The average number of inpatient visits was 2.83, with a mean LOS of 14.69 days. In addition, 78.3% of the patients had ≥1 ER visit with a mean of 6.20 visits and a median of 3 visits. Also, 94.5% had ≥1 outpatient hospital visit (mean: 13.63 visits; median: 9 visits). About 86.5% had ≥1 outpatient office visit (mean: 8.06 visits; median: 5 visits) during the 12-month follow-up period (Fig. 6).

The mean LOS for inpatient stays was 13.35 days for SCD-related hospitalization. Furthermore, 64% of the patients had ≥1 SCD-related hospitalization and the mean number of visits was 2.5. About 67.3% of the patients had ≥1 SCD-related outpatient ER visit, with a mean number of 4.71 visits and median of 2 visits. About 64.3% of the patients had ≥1 outpatient office visit with a mean of 3.86 and median of 2 visits. Also, 89.8% of patients had ≥1 SCD-related outpatient hospital visit, with a mean of 9.97 visits and median of 7 visits during the 12-month follow-up period (Fig. 6).

#### Health care costs

**Fig. 7:**
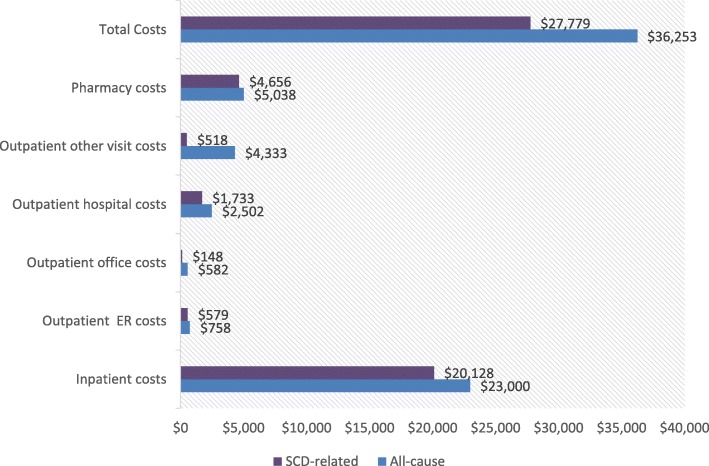
All cause and SCD-related health care costs. SCD: sickle cell disease; ER: emergency room

Mean total all-cause health care costs during the 12-month follow-up period were $36,253 and the majority were due to inpatient costs ($23,000). In addition, pharmacy costs were $5038, outpatient costs were $7417, ER costs were $758, and other costs including laboratory visit and ambulatory costs, amounted to $40. SCD-related health care costs during the 12-month follow-up period were $27,779 (mean total costs), and the main cost driver was inpatient costs ($20,128). Pharmacy costs were $4656, outpatient costs were $2399, ER costs were $579, other costs, which included laboratory visits and ambulatory costs, amounted to $18 (Fig. 7).

## Discussion

This study provides real-world evidence of the demographic and clinical characteristics, treatment patterns, and economic burden of adult and pediatric SCD patients who were prescribed HU treatment. Although HU was only recently approved for pediatric use (December 2017), the results of this study showed that nearly half the study population of patients prescribed HU were aged under 18 years.

Although studies have shown that continuing regular HU treatment decreases or prevents complications of SCD [34], our study showed poor adherence to HU treatment. Specifically, findings included a mean MPR of 0.52, less than half of the study population had an MPR ≥50%, and less than one-quarter of patients had an MPR ≥80%. The results are similar to the findings in another study that observed low HU adherence, in which 38% of SCD patients with HU treatment had an MPR ≥80% [35]. In addition, defining HU discontinuation using a 30- to 90-day prescription gap between two subsequent prescriptions, the rate of HU discontinuation ranged from 58.9 to 87.8%, and 52.5 to 76.7%, among those who reinitiated HU. Specific reasons for discontinuation or poor HU adherence could not be ascertained in this study. However, a previous study carried out among younger SCD patients (aged 12–18 years) showed that half the participants had low HU adherence, and that patients who were more concerned about HU-related adverse events were more likely to report lower HU adherence [36]. Other studies have shown that, in addition to concerns such as HU-related adverse events, the costs and inconvenience associated with monitoring required during HU therapy as well as recall barriers and forgetfulness may contribute to the low HU compliance observed in most patients [33, 37, 38]. Furthermore, Badawy et al. showed that patients’ concerns about potential adverse events associated with HU, and concerns about overuse of HU medication in general, were associated with worse health-related quality of life [36]. A survey study of over 200 pediatric hematologists managing SCD patients revealed that the most common factors identified as barriers to HU use involved compliance. Most health care providers acknowledged that concerns with low HU compliance were low laboratory monitoring compliance, and low contraception compliance among female patients [39]. Furthermore, over 99% of the SCD patients in this study had a form of complication associated with VOC, and the rate of ACS among study patients was 10.13%. Although few studies have examined the rate of ACS in SCD patients, to our knowledge, no other study has examined the rate of ACS among SCD patients who were prescribed HU.

In addition to HU adherence, our study also examined monitoring patterns of SCD patients with HU use. The HU treatment guidelines for SCD patients recommend monitoring at least every 8–12 weeks. Evidence suggests that SCD patients treated with HU who received frequent monitoring had improved medication adherence and clinical outcomes [40]. A study showed monitoring of SCD patients on HU occurred every 4–6 weeks during escalation of therapy and every 8–12 weeks upon achievement of maximal tolerated dose (MTD). Monitoring included a physical examination, patient’s history, and laboratory testing. The most commonly ordered laboratory tests used to monitor SCD patients on HU were complete blood counts, although liver function tests, renal function tests, and reticulocyte count are also frequently monitored [41]. However, this study’s results showed that only 73.6% of SCD patients prescribed HU had ≥1 complete blood count test post-HU initiation, 27.2% of the patients had ≥1 serum iron study done, and the mean number of laboratory visits was 1.3 during the 12-month follow-up period.

This study also observed a high rate of morbidity associated with SCD, as a large proportion of all-cause hospitalizations (98.4%), LOS (90.9%) and ER admissions (86%) were attributed to SCD. An ad hoc analysis carried out assessing Medicare SCD patients with or without a history of HU use showed that 85.8% of the LOS for all-cause hospitalization and 82.9% of inpatient stays were attributed to SCD-related diagnoses. These results are similar to those from other studies that have analyzed SCD-related health care utilization, in which SCD patients were observed to have had an average of 3.7 inpatient hospitalizations and 24.1 hospital days, with roughly 84% attributable to SCD-related diagnoses during the study period [16]. Also, SCD patients incurred higher costs (76.6% of total health care cost attributed to SCD-related diagnoses). The results are again similar to the study by Kauf et al. that found 64% of costs were SCD-related [16]. HU adherence among SCD patients has also been associated with decreased risk of SCD-related hospitalization, all-cause and SCD-related ER visits, and vaso-occlusive events [32, 42]. In addition, the difference in all-cause health care resource utilization in the baseline and follow-up periods showed a decrease in all-cause hospitalization, inpatient LOS, and outpatient ER visits. The decrease may have been due to the reduction in severe SCD-related complications that would warrant ER and inpatient visits, or longer hospitalizations possibly resulting from HU utilization during the follow-up period.

Few studies have examined the proportion of SCD patients who are prescribed HU as well as pain medication. Our study found that almost half of the patients were prescribed opioids (49.0%) and nonsteroidal anti-inflammatory drugs (NSAIDs) (55.5%) during the follow-up period. Another study showed that older age, HU therapy, NSAID use, and frequent inpatient hospitalizations were associated with high-dose opioid use [43]. The high rate of pain medication utilization in this population of SCD patients may be because these SCD patients have a higher tendency to use pain medication because of high disease morbidity. Recommendations for HU use include SCD patients with ≥3 moderate to severe pain episodes within a 12-month period [22, 44, 45]. This study also observed a low rate of indications for pneumococcal and meningococcal vaccines during the 12-month follow-up period (4.4 and 2.9%, respectively).

The abovementioned results on treatment patterns for vaccines are limited in that they may not have captured the true rate of SCD patients prescribed HU who were also administered these vaccines, since they are not given annually. It is possible that most patients received the recommended vaccine prior to the 12-month follow-up period, or their next vaccine dose may have occurred outside the study’s 12-month follow-up period [46].

More generally, certain other limitations are associated with the use of any claims data, which are collected for the purpose of payment and not research. While medication adherence was measured using MPR, which is a widely accepted method for measurement in observational studies, the presence of a claim for a filled prescription does not indicate whether the medication was taken as prescribed or at all. Moreover, medications filled over-the-counter or provided as samples by the physician are not observed in claims data. In addition, the presence of a diagnosis code on a medical claim does not indicate a positive presence of disease, as the diagnosis code may be incorrectly coded or included as rule-out criteria rather than actual disease. Finally, claims data cannot capture certain demographic and clinical parameters.

For this study, results related to medication use and HU treatment patterns, among other results, may not be generalizable to other populations. The study setting is within the Medicaid population that consists of patients with disabilities, children of low-income families, pregnant women, parents of Medicaid-eligible children who meet certain income requirements, and low-income seniors [47]. These populations are more likely to have unmet needs in health care resource services. Individuals with dual eligibility for both Medicaid and Medicare were excluded, since data for these observations are not complete; together with the likelihood that many patients switched entirely from Medicaid to Medicare at age 65, this exclusion may have resulted in underrepresentation of patients aged 65 years or older, and therefore further limits the generalizability of the results to broader populations. Additionally, due to the limited availability of the data, the data of managed care plan patients only includes 14 states, and the study period until December 2013 was the most recent data at the time of study. SCD management guidelines have remained consistent in the past decade, so the findings of this study should remain valid in the current year. Furthermore, health care utilizations and costs can only be identified among patients enrolled in an FFS health plan. Therefore, the actual health care costs may be higher or lower than reported here, which may limit the ability of cost outcomes to be generalized to the entire Medicaid SCD population.

Finally, it is important to note that the present study was limited in scope to general associations between treatments and economic outcomes, and does not include certain parameters that may affect outcomes, such as associations between compliance and age and comorbidities, clinical guidelines, and certain clinical parameters that fell out of scope; nonetheless, the findings underscore the need for future investigation of more specific research questions.

## Conclusions

Despite the positive disease-modifying effects of HU therapy, SCD patients treated with HU continue to have significant unmet needs in terms of medication adherence, high rates of treatment discontinuation, and high economic burden. Future research is needed to evaluate the reasons for discontinuation in a real-world data population, and to develop disease management strategies to help alleviate the SCD-related health care burden.

## Supplementary information

**Additional file 1: Table S1.** Sensitivity Analysis for Discontinuation Using 30- and 60-Day Refill Gaps.

## Data Availability

All data sets supporting the conclusions of this article are included within the article (and its additional files).

## Abbreviations

ACS: Acute chest syndrome
CCI: Charlson comorbidity index
CT: Computerized tomography
ER: Emergency room
FFS: Fee for service
HCUP: Healthcare Cost and Utilization Project
HU: Hydroxyurea
ICD-9-CM: International Classification of Diseases, Ninth Revision, Clinical Modification
LOS: Length of stay
MAX: Medicaid Analytic Extracts
MOA: Mechanism of action
MPR: Medication possession ratio
NSAID: Nonsteroidal anti-inflammatory drug
SCD: Sickle-cell disease
TCD: Transcranial doppler
VOC: Vaso-occlusive crisis
