# Supplementary material for: Treatment patterns and economic burden of sickle-cell disease patients prescribed hydroxyurea: a retrospective claims-based study
Source: Health Qual Life Outcomes. 2019 Oct 16;17:155. doi: 10.1186/s12955-019-1225-7 (PMC6794855; doi:10.1186/s12955-019-1225-7)
Supplement: Supplementary file 1 — Additional file 1: Table S1. Sensitivity Analysis for Discontinuation Using 30- and 60-Day Refill Gaps. [file 12955_2019_1225_MOESM1_ESM.docx]

**Table S1. Sensitivity Analysis for Discontinuation Using 30- and 60-Day Refill Gaps**

| **HU Discontinuation (60-day Gap)** | **N/mean** | **%/SD** |
| --- | --- | --- |
| Discontinuation | 2,878 | 72.0% |
| Time-to-discontinuation (days) | 165.50 | 137.24 |
| Re-initiation (among patients who discontinued N=2,878) | 1,870 | 65.0% |
| **HU Discontinuation (30-day Gap)** |  |  |
| Discontinuation | 3,512 | 87.8% |
| Time-to-discontinuation (days) | 113.95 | 113.91 |
| Re-initiation (among patients who discontinued N=3,512) | 2,692 | 76.7% |

HU: hydroxyurea; SD: standard deviation
